# Editorial: Advances in Head and Neck Cancer Immunology and Immunotherapy

**DOI:** 10.3389/fonc.2019.00172

**Published:** 2019-03-19

**Authors:** Rasha Abu Eid

**Affiliations:** ^1^Institute of Dentistry, School of Medicine, Medical Sciences and Nutrition, University of Aberdeen, Aberdeen, United Kingdom; ^2^Institute of Medical Sciences, School of Medicine, Medical Sciences and Nutrition, University of Aberdeen, Aberdeen, United Kingdom

**Keywords:** head and neck cancer, cancer immunology, immunotherapy, immune checkpoint inhibitors, cancer vaccines, regulatory T cells, human leukocyte antigen

Head and neck cancers are a group of malignancies that affect the head and neck region. These include cancers of the oral cavity, oropharynx, larynx, lips, sinuses, and nasal cavity. The incidence of head and neck cancer is on the rise which is mainly attributed to changes in risk factors including increased alcohol and tobacco consumption and betel chewing habits in certain parts of the world. Additionally, human papilloma virus (HPV) infections have contributed to the rise of head and neck cancers, in particular oropharyngeal cancers, especially in younger patients.

Despite advances in cancer diagnosis and treatment, the mortality rate of head and neck cancer remains unchanged. The high mortality rate is mainly attributed to late diagnosis, and the lack of effective treatments for late stage cancers.

Cancer immunotherapy is among the greatest advances in cancer therapy. Various treatment modalities that target different components of the immune system have proven successful in controlling disease progression and even producing long lasting cures in some types of cancer. Cancer immunotherapy's biggest successes were reported in melanoma and lung cancer. However, many other cancer types are benefiting from the advances in cancer immunotherapy including head and neck cancer. In fact, several immunotherapies have been approved for the treatment of head and neck cancer, including immune checkpoint inhibitors for the management of recurrent or metastatic cancers.

Although the success of cancer immunotherapy cannot be disputed, many patient responses are transient and short lived. This can be explained by different immune escape mechanisms deployed by cancer cells including dampening the immune response through modulating immune checkpoints, in addition to the recruitment and *de novo* differentiation of suppressive immune cells such as regulatory CD4 T cells.

In this research topic, two review articles (Forster and Devlin; Ward et al.) discussed the use of immune checkpoint inhibitors in the treatment of head and neck cancer.

Forster and Devlin provided a review of different co-inhibitory and co-stimulatory checkpoints that could be targeted by immune checkpoint inhibitors in the context of immunotherapy. Their article included a review of the role of the PD-1/PDL-1 axis and GITR in cancer immunology and immunotherapy. The review article by Forster and Devlin presented a comprehensive and exciting review of different therapeutic combinations with checkpoint inhibitors, including different immune modulators, viral therapies, and chemoradiotherapy. The review then summarized the adverse effects associated with immune checkpoint inhibitor therapy and highlighted the importance of biomarkers for the prediction of disease progression and response to therapy.

In their manuscript, Ward et al. provided an overview of the timeline for FDA approvals of different immune checkpoint inhibitors in the treatment of different cancers. The manuscript detailed the history, function, and application of anti-CTLA-4, anti-PD-1, and anti-PDL-1 antibodies in cancer immunotherapy before providing an in depth review of the use of immune checkpoint inhibitors in head and neck cancer. The manuscript by Ward et al. provided an interesting review of possible modifications of existing checkpoint inhibitors including antibodies that target a soluble isoform of CTLA-4 (sCTLA-4).

The presence of suppressive immune cells in the tumor microenvironment, and in particular regulatory CD4 T cells, has been shown to adversely affect the patient prognosis and the anti-tumor immune response. Several strategies are under study to selectively target these suppressive cells as part of cancer immunotherapy. However, there is some conflicting evidence in the literature regarding the role of regulatory CD4 T cells in head and neck cancer. The systematic review by O'Higgins et al. investigated this controversy and systematically analyzed the available evidence. Their findings revealed a deficiency in fully characterizing regulatory T cell phenotypes in the studied head and neck tumors, especially with regard to HPV status, which could contribute to the discrepancy in describing the role of regulatory CD4 T cells in tumor progression. Furthermore, the findings of the systematic review by O'Higgins et al. uncovered a real need for developing robust markers for phenotyping T cells and for detecting regulatory CD4 T cells systemically and within the tumor microenvironment.

HPV positive head and neck cancers represent a particularly appealing target for cancer immunotherapy because of their intrinsic immunogenicity. In addition to the immune response mounted in response to the virus itself, tumors positive for HPV over-express E6 and E7 which can be recognized by the immune system as non-self antigens and can therefore be ideal targets for vaccine based immunotherapies. In their review article, Wang et al. discussed the use of cancer vaccines in the prevention and the treatment of head and neck cancer. They discussed the differences between HPV positive and HPV negative tumors and provided a comprehensive review into various target antigens (viral antigens, neoepitopes, and tumor associated antigens) and different vaccine platforms (DNA, mRNA, peptide, viral, bacterial vector, and cellular vaccines).

Measures for predicting patient survival in head and neck cancer are still lacking. Wichmann et al.'s original research article reported the potential use of a Human Leucocyte Antigen (HLA) score to predict progression free survival in head and neck cancer patients. In their study, Wichmann et al. used HLA traits known to be predictors of progression free survival to build a scoring system using genetic information from HLA typing for predicting prognosis. The findings of their study have significant clinical potential for predicting relapse and for the stratification of patients for clinical trials and informing personalized treatment.

Given the complexity of the immune response to cancer, no single therapeutic agent is capable of enhancing the effector arms of the immune response while simultaneously targeting the suppressive arm. The collection of articles in this research topic suggests a role for combination therapies in the treatment and management of head and neck cancer. Many clinical trials are ongoing that are testing various combinations of modulators of the immune system for the management of head and neck cancer.

The combination of diverse immunotherapies that target different arms of the immune response is gaining acceptance in the clinical setting and could potentially provide a solution for sustaining short lived anti-tumor immune responses.

## Author Contributions

The author confirms being the sole contributor of this work and has approved it for publication.

### Conflict of Interest Statement

The author declares that the research was conducted in the absence of any commercial or financial relationships that could be construed as a potential conflict of interest.

